# Extracellular Vesicle-Associated miRNAs in Glioblastoma: Mechanisms, Biomarkers, Therapies, and Links to Neurodegeneration

**DOI:** 10.3390/cancers18081269

**Published:** 2026-04-16

**Authors:** Chun Li, Takahiro Ochiya

**Affiliations:** Department of Molecular and Cellular Medicine, Institute of Medical Science, Tokyo Medical University, 6-7-1 Nishishinjuku, Shinjuku-ku, Tokyo 160-0023, Japan; ri.shun.6y@tokyo-med.ac.jp

**Keywords:** extracellular vesicles, miRNA, glioblastoma, liquid biopsy, biomarker, blood–brain barrier, therapeutic strategies, neurodegenerative diseases

## Abstract

Extracellular vesicles (EVs) facilitate intercellular communication in glioblastoma (GBM) by transferring microRNAs (miRNAs). GBM is the most aggressive primary brain tumor in adults, and despite multimodal therapy, the median survival remains approximately 15 months. Current diagnostic approaches, including contrast-enhanced MRI, are insufficient to reliably distinguish true tumor progression from pseudoprogression. Moreover, therapeutic efficacy is limited by intratumoral heterogeneity, acquired resistance, and the restrictive nature of the blood–brain barrier (BBB). In this context, EV-associated miRNAs (EV-miRNAs) contribute to GBM progression by regulating proliferation, angiogenesis, invasion, therapeutic resistance, and immune evasion. Notably, several EV-miRNAs are dysregulated in both GBM and neurodegenerative diseases (NDDs), suggesting shared molecular pathways across central nervous system (CNS) disorders. Circulating tumor-derived EV-miRNAs represent promising liquid biopsy biomarkers for diagnosis, prognosis, and longitudinal treatment monitoring. Beyond their biomarker potential, EVs can be engineered as nanocarriers capable of crossing the BBB to deliver therapeutic cargo, including inhibitors of oncogenic miRNAs (e.g., miR-21) or tumor-suppressive miRNAs (e.g., miR-124). This review summarizes the molecular functions, biomarker applications, and therapeutic strategies of EV-miRNAs in GBM. We further discuss current challenges related to methodological standardization, scalable production, and clinical translation. Collectively, advancing the understanding and clinical implementation of EV-miRNAs may provide new opportunities for precision diagnostics and therapeutic innovation in GBM.

## Simple Summary

Glioblastoma (GBM), the most lethal adult brain cancer, has a median survival of only ~15 months despite aggressive treatment. MRI struggles to distinguish true progression from treatment effects, whereas tumor heterogeneity, resistance, and the blood–brain barrier (BBB) hinder therapy. Extracellular vesicles (EVs) carry microRNAs (miRNAs) that drive GBM progression and are also altered in neurodegenerative diseases (NDDs). Circulating EV-miRNAs may enable simple blood- or CSF-based liquid biopsies. Engineered EVs provide improved BBB penetration to deliver oncomiR inhibitors or tumor-suppressive miRNA mimics. This review covers their biology, biomarker utility, therapeutic promise, and clinical challenges.

## 1. Introduction

Extracellular vesicles (EVs), nano-sized particles surrounded by a lipid bilayer, are secreted by almost all cells [1]. EVs are loaded with bioactive molecules, including lipids, proteins, and microRNAs (miRNAs), which reprogram the functions of recipient cells under normal physiology and in disease [1,2]. EVs have been a topic of interest in the last decade as key messengers for cell-to-cell communication in the central nervous system (CNS) [3]. In the CNS, EVs play an active role in communication between neurons and neighboring cells, such as astrocytes, microglia, and oligodendrocytes [4]. EVs cross the blood–brain barrier (BBB), although this varies by EV subtype and source, and play critical roles in cerebral homeostasis and pathology [5]. Brain-derived EVs contribute to neural development, synaptic function, and the pathogenesis of brain tumors and neurological diseases; therefore, research on them has expanded rapidly [6,7].

miRNAs, a key EV cargo, have attracted interest because of their powerful post-transcriptional gene regulation abilities. These short noncoding RNAs modulate target mRNAs by base pairing with their 3′ untranslated regions (3′-UTRs) [8,9]. One miRNA can regulate hundreds of targets, whereas a single gene receives inputs from multiple miRNAs [10]. EV lipid bilayers protect miRNAs from RNase degradation in biofluids, enabling mechanistic investigations and therapeutic applications [11,12].

EV-associated miRNAs (EV-miRNAs) regulate CNS physiology and pathology, thereby functioning as key mediators of glioblastoma (GBM) progression [7,13]. GBM is the most lethal brain tumor in adults and accounts for approximately 50% of all gliomas [14]. Standard treatment, which includes maximal safe resection, radiotherapy, and temozolomide (TMZ) chemotherapy, yields a median survival of approximately 15 months and a 5-year survival rate of less than 5% [14]. Intratumoral heterogeneity, rapid onset of therapy resistance, inadequate BBB penetration, and the inability of conventional imaging to distinguish true progression from pseudoprogression/radiation necrosis remain the key reasons behind these unfavorable results [15,16]. miRNAs act as either oncomiRs that promote tumor growth or as tumor suppressors that inhibit tumor growth [17]. EVs can be engineered as therapeutic carriers to deliver miRNAs for GBM treatment [7,18]. Interestingly, altered EV-miRNA profiles not only characterize GBM progression but also neurodegenerative diseases (NDDs) [19,20]. The shared dysregulation of key EV-miRNAs, such as miR-21 and miR-124, across GBM and NDDs may suggest partially overlapping molecular pathways linking these distinct CNS pathologies [20].

In addition to their mechanistic roles, EV-miRNAs hold great potential as biomarkers for GBM diagnosis, prognosis prediction, and therapeutic monitoring [21,22]. Tumor-derived EVs cross the BBB and are detectable in the peripheral blood or cerebrospinal fluid (CSF) as liquid biopsy-based biomarkers of miRNA content [21,22,23]. These features, as well as the stability of EV lipid bilayers, imply that EV-miRNAs may have clinical utility in liquid biopsies for GBM. Currently, research strategies involve miRNA sponge-mediated inhibition of oncomiRs (e.g., miR-21) and restoration of tumor-suppressive miRNAs (e.g., miR-124), as well as EV engineering to enhance CNS delivery efficiency [23,24]. This could help overcome the drug-delivery limitations of conventional GBM treatment methods.

This review offers a broad perspective on EV-miRNAs in GBM from five aspects: (1) the production, sorting, secretion, and uptake of EV-miRNAs; (2) the mechanisms of EV-miRNAs in GBM, including oncomiRs, tumor suppressors, and their overlap with NDDs (Table 1); (3) liquid biopsy-based biomarkers for diagnosis, prognosis, and monitoring (Table 2); (4) therapeutic approaches and designed delivery vehicles; and (5) challenges and future perspectives.

## 2. EV-miRNAs: Biogenesis, Secretion, and Uptake

EV-miRNAs undergo highly regulated biogenesis, selective cargo loading, and exocytic release [61]. miRNA sorting into EVs is influenced by sequence-specific motifs and protein factors derived from the tumor microenvironment [7,62].

### 2.1. Biogenesis and Selective Sorting of EV-miRNAs

Conventional processing generates the miRNA pool that is available for loading into EVs. Pri-miRNAs are cleaved by the nuclear Drosha/DGCR8 complex into pre-miRNAs, and cytoplasmic Dicer further processes them into mature miRNAs. These miRNAs bind to Argonaute (AGO) proteins to form the RNA-induced silencing complex (RISC), and a subset is then selectively sorted into EVs [11,63].

Different subtypes of EVs exist, including exosomes (typically 30–150 nm and classically considered of endosomal origin), microvesicles (100–1000 nm arising from plasma membrane budding), and apoptotic bodies (500–5000 nm released during apoptosis containing cellular organelles) [1]. All EV subtypes transfer miRNAs to target cells. Given that these subtypes exhibit a strong overlap in their physical properties, differentiation between them is challenging. The International Society for Extracellular Vesicles (ISEV) has proposed size-based (small/medium/large EVs) or marker-based (e.g., CD9, CD63, and CD81) classifications [64].

Accordingly, EV-miRNA profiles are distinct from cellular miRNA abundance, demonstrating selective partitioning. This process is mediated by RNA-binding proteins (hnRNPA2B1, YBX1, and MEX3C), which recognize conserved motifs in target miRNAs [11,62,65]. Interactions between proteins and RNAs, such as protein–protein and protein–RNA interactions, help incorporate cargo into nascent vesicles. EXO motifs drive vesicular export, whereas CELL motifs promote cytoplasmic retention [62,65]. This selective enrichment results in tumor-derived EV-miRNA signatures enriched for oncogenic miRNAs (e.g., miR-21 in GBM) [7].

### 2.2. EV-miRNA Secretion and Uptake Mechanisms

Distinct cellular pathways regulate EV secretion. Exosomes are generated by the fusion of multivesicular bodies with the plasma membrane, and microvesicles arise from the direct outward budding of the plasma membrane. For example, hypoxia and HIF-1α activation stimulate EV generation and alter miRNA content, thereby linking the EV–miRNA profile to the GBM microenvironment [66]. EVs are taken up by recipient cells through endocytosis or direct membrane fusion [67].

Once internalized, miRNAs associate with RISC complexes in recipient cells, thereby modulating gene expression and influencing key oncogenic pathways [11,63]. Quantitative stoichiometric analysis has demonstrated that the mean miRNA copy number per exosome is approximately 0.00825 molecules, meaning that more than 100 individual EVs would need to be internalized to deliver a single copy of a given miRNA to a recipient cell [68]. This sub-stoichiometric reality raises questions about the physiological relevance of endogenous EV-miRNA signaling and remains actively debated. It is therefore important to note that therapeutic applications employing engineered EVs are generally considered to operate in a distinct pharmacological context, where deliberate cargo enrichment may achieve miRNA concentrations that differ substantially from those of naturally secreted vesicles. Although uptake mechanisms have been relatively well studied, the quantitative aspects of EV-miRNA signaling via EVs warrant further investigation.

## 3. The Roles of EV-miRNAs in GBM

EV-miRNAs regulate multiple aspects of GBM pathobiology (Table 1). Table 1 summarizes selected EV-miRNAs discussed in this review and is not intended to be exhaustive; readers are referred to Singh et al. [7] and Dai et al. [66] for more comprehensive listings of GBM-associated EV-miRNAs. In GBM, EV-miRNAs can function as either oncomiRs or tumor suppressors to regulate tumor progression [7]. Some EV-miRNAs, such as miR-21 and miR-124, are also dysregulated in NDD-related contexts [20].

### 3.1. Oncogenic Functions of EV-miRNAs

miR-21 is one of the most well-studied oncomiRs in several malignancies, including GBM [17]. CSF EV-miR-21 is elevated in patients with GBM [25] and is associated with poor prognosis and tumor recurrence [55]. EV-miR-21 derived from glioma stem cells promotes endothelial cell proliferation and angiogenesis via VEGF/VEGFR2 signaling, as demonstrated in vitro [26]. Under hypoxic conditions, glioma-derived exosomal miR-21 promotes myeloid-derived suppressor cell (MDSC)-mediated immunosuppression by inhibiting PTEN and activating the PI3K/AKT pathway in vitro, with these MDSC-mediated immunosuppressive effects further confirmed in vivo in mouse glioma models [27]. EV-miR-21 from glioma-associated microglia/macrophages (GAMs) exacerbates TMZ resistance via STAT3 activation in vitro [28].

miR-221, which is frequently enriched in glioma-derived EVs, targets DNM3 (a GTPase involved in endocytosis and membrane remodeling) to induce tumor progression and TMZ resistance in recipient cells [33]. Similarly, EV-miR-1246 targets TERF2IP (a telomere-associated protein and NF-κB co-activator) to activate STAT3 signaling and suppress NF-κB activity, thereby promoting M2 macrophage polarization under hypoxic conditions [34]. CSF- and plasma-derived exosomal miR-1246 further drives MDSC differentiation and activation, thereby strengthening GBM immune evasion and tumor proliferation [35].

In addition to immune modulation, EV-miRNAs also drive glioma migration and invasion. EV-miR-10b-5p and miR-1246 are abundant in hypoxic glioma-derived EVs and promote cell migration and invasion in recipient normoxic glioma cells by targeting TFAP2A and FRK, respectively, in vitro [36].

The effects of EV-miRNAs extend beyond those of individual miRNAs. Multiple EV-associated miRNAs may act cooperatively to modulate recipient cells [36]. These interactions may create complex regulatory networks in the GBM microenvironment, contributing to its aggressive biology [7]. A thorough analysis of EV-miRNA networks is warranted, as co-targeting critical oncomiRs has been reported to more effectively inhibit tumor cell proliferation, invasion, and drug resistance than single-miRNA targeting in preclinical models [69,70], although evidence for EV-specific co-delivery remains limited.

### 3.2. Tumor-Suppressor Functions of EV-miRNAs

EV-miRNAs act as tumor suppressors in GBM by inhibiting proliferation, migration, and angiogenesis, or by enhancing treatment sensitivity. miR-124 is a well-studied tumor-suppressive miRNA that is downregulated in GBM compared to that in normal brain tissue [37,38]. In a three-dimensional (3D) microfluidic GBM model in vitro, EV-miR-124 suppressed GBM cell proliferation and migration, as shown by reduced STAT3 expression [39]. EV-miR-124 also shifts microglia from the M2 (pro-tumoral) to the M1 (anti-tumoral) phenotype, associated with altered STAT1/STAT3 balance, primarily via STAT3 suppression, thereby potentially strengthening antitumor immunity in the TME [39].

In preclinical studies, exosomes derived from Wharton’s jelly MSCs delivered exogenous miR-124 to GBM cells, reduced CDK6 (a cell cycle kinase promoting G1-S phase progression) expression, and enhanced TMZ sensitivity [40]. Systemic administration of BM-MSC-derived exosomes engineered to overexpress miR-124a resulted in 50% long-term survival at 110 days in mice bearing intracranial GSC267 (glioma stem cell 267) xenografts, compared with no long-term survivors in the control group; histological analysis confirmed no residual tumor in survivors [41]. Mechanistically, miR-124a silences FOXA2, a pro-tumorigenic transcription factor and intermediary of lipid metabolism in GSCs, leading to aberrant intracellular lipid accumulation [41], highlighting the strong therapeutic potential of EV-delivered miR-124a (Section 5).

miR-1 is significantly downregulated in GBM patient samples compared with normal brain tissue [53,60]. By directly targeting ANXA2 (Annexin A2, an important pro-oncogenic protein), loading miR-1 into GBM-derived EVs reduced invasion, neurosphere formation, and endothelial tube formation in vitro [53]. miR-199a is downregulated in glioma tissues and cells, with concurrent overexpression of its target AGAP2 (Arf GTPase-activating protein-2, an oncogenic protein); MSC-derived exosomes delivering miR-199a suppressed glioma cell proliferation, migration, and invasion by downregulating AGAP2 in vitro [54].

These findings highlight the importance of restoring tumor-suppressive EV-miRNAs in GBM, although most supporting evidence remains preclinical, and optimal dosing regimens, effective miRNA combinations, and improved delivery systems still require further investigation [71].

### 3.3. Shared EV-miRNA Dysregulation in GBM and NDDs

This section focuses on EV-miR-21 and EV-miR-124, which are among the most extensively studied and functionally validated EV-miRNA candidates linking GBM to NDD-related contexts [20]. EV-miR-21 is discussed mainly in relation to Alzheimer’s disease (AD) and Parkinson’s disease (PD), whereas EV-miR-124 is additionally considered in the context of amyotrophic lateral sclerosis (ALS) [20,72]. These EV-miRNAs influence shared cellular processes, particularly microglial reprogramming and inflammatory signaling.

For example, EV-miR-21 is highly abundant in GBM-derived EVs [73]. In AD-related systems, it is elevated in SH-Swe neuronal exosomes, derived from an SH-SY5Y neuronal model expressing Swedish mutant APP, and propagates through a neuron–microglia EV shuttling chain in vitro [29]. In PD, serum EV-miR-21 is downregulated in patients with progressive motor and cognitive decline [30]; this represents a correlative biomarker association, and direct EV-specific mechanistic evidence in PD remains absent.

Functionally, glioma-derived EVs transfer miR-21 primarily to microglia in vitro and in vivo [73,74], where it downregulates Btg2 (an antiproliferative tumor suppressor that restrains cell cycle progression at the G1/S checkpoint), promoting microglial reprogramming towards a tumor-permissive phenotype and thereby facilitating GBM progression [74]. In vitro, SH-Swe neuronal exosomes enriched in miR-21 induce pro-inflammatory activation in CHME3 human microglia, modeling exosome-driven neuroinflammation in AD [29]; however, this remains an in vitro finding, and causal EV-miR-21-driven neuroinflammation has not been demonstrated in human AD. At the cellular level, miR-21 is upregulated in MPP^+^-treated dopaminergic neurons (in vitro PD model), and its inhibition reduces apoptosis and pro-inflammatory cytokine production [31]; whether this extends to EV-mediated signaling remains unknown.

Therapeutically, in GBM, engineered exosomes delivering a miR-21 sponge suppressed proliferation and induced apoptosis in GBM cells in vitro and reduced tumor volume in a rat xenograft model [75]. In an AD mouse model, hypoxia-preconditioned MSC-derived exosomes reduced amyloid-β burden and neuroinflammation in APP/PS1 mice, with miR-21 identified as an important contributing cargo [32]. However, no robust miR-21-specific knockout or overexpression data in human AD are currently available, and comparable EV-miR-21-specific therapeutic evidence in PD is still lacking.

EV-miR-124 exhibits a similarly complex cross-disease expression pattern. In GBM, miR-124 is downregulated within tumor cells and acts as a tumor suppressor, as discussed in Section 3.2. Paradoxically, serum EV-miR-124-3p is elevated in GBM patients compared with healthy controls [56,57], suggesting preferential sorting into circulating EVs, although the mechanism remains unclear. In contrast, EV-miR-124-3p has been reported to be reduced in serum and CSF EVs from AD patients [42], consistent with its loss in brain tissue and with reported roles in AD-related pathways, including modulation of the APP/BACE1 axis [43], and regulation of tau phosphorylation [44]. In PD, total circulating miR-124 levels are reduced [45]; however, dedicated patient-biofluid evidence specifically for EV-miR-124 in PD remains limited. miR-124 is reduced in the spinal cord of end-stage SOD1G93A ALS mice [46,47], while its association with motor neuron-derived EVs paradoxically increases, suggesting a disease-associated active exosomal sorting of miR-124-3p out of degenerating neurons [47].

Functional evidence for EV-miR-124 in pathological contexts is derived primarily from rodent and in vitro models. Microglia-secreted small EVs modify tumor growth in a murine GL261 glioma model by transporting miR-124 to both astrocytes and glioma cells, enhancing glutamate clearance and suppressing tumor cell metabolism, respectively [76]; human validation data for this endogenous microglial EV-miR-124 signaling are lacking. In AD, SH-Swe neuronal exosomes, which contain miR-124 among other miRNAs, are internalized by CHME3 microglia and induce pro-inflammatory activation in vitro; however, the specific contribution of miR-124 remains unresolved [29]. Garcia et al. [48] further showed that neuronal miR-124 can reshape microglial plasticity, with exosomes contributing substantially to its transfer from APP-Swe neuronal cells to activated microglia. In ALS, exosomes derived from hSOD1-G93A-transfected NSC-34 motor neurons were enriched in miR-124 and, upon transfer to N9 microglia in vitro, induced sustained NF-κB activation, pro-inflammatory cytokine upregulation, and a 50% reduction in phagocytic capacity—implicating mutant motor neuron-derived EV cargo, potentially including miR-124, in pathological microglial remodeling [49]. Direct functional evidence for endogenous EV-miR-124 in PD patient-derived systems remains limited; however, the therapeutic use of exogenously loaded EV-miR-124-3p has been explored, as discussed below.

Therapeutic use of exogenous EV-miR-124 has been explored across multiple disease models. Engineered EVs enriched in miR-124 suppressed GBM cell proliferation and M2 microglial polarization in a 3D microfluidic tumor model in vitro, demonstrating the anti-tumorigenic potential of exogenous EV-miR-124 delivery [39]. More recently, engineered miR-124-3p-enriched exosomes have shown therapeutic potential in AD models, including neuro- and immunoprotective effects in a microfluidic neuron–glia triculture platform and improved cognition with reduced neuroinflammation in an AD mouse model [50,51]. In PD, externally loaded miR-124-3p-enriched EVs derived from umbilical cord blood mononuclear cells (UCB-MNCs) protected dopaminergic neurons in a 6-OHDA mouse model [52]. Currently, direct therapeutic evidence for exogenous or engineered EV-miR-124 in ALS remains lacking.

Taken together, available studies suggest that EV-miR-21 and EV-miR-124 can influence microglial state in a context-dependent manner, with donor-cell identity likely shaping the biological outcome. However, their precise roles in GBM–NDD crosstalk remain poorly understood, as most functional evidence derives from rodent or in vitro systems, and EV-specific mechanistic data in human patient-derived contexts remain scarce. Integrated single-cell transcriptomic and EV-miRNA profiling approaches may help better characterize their cell-type-specific and context-dependent functions.

## 4. EV-miRNAs as Liquid Biopsy Biomarkers in GBM

EV-miRNAs provide a stable liquid biopsy source of tumor-associated markers in GBM [77,78]. EV-miRNA profiling offers a less invasive and potentially repeatable molecular readout of tumor status [78], which may complement serial imaging and tissue biopsy. Methodological design, EV isolation/characterization, and reporting of EV-miRNA biomarker studies should adhere to the MISEV2023 guidelines [64], with biofluid-specific considerations for blood and CSF EVs [79,80,81].

### 4.1. Diagnostic Biomarkers

The diagnostic potential of EV-miRNAs is well illustrated by CSF-derived EV-miR-21. For instance, CSF EV-miR-21 levels were approximately 10-fold higher in patients with GBM than in non-tumor controls and distinguished GBM from controls with an AUC of 0.91, a sensitivity of 87%, and a specificity of 93% [25]. CSF EV-miR-21 may serve as a promising biomarker for supporting diagnosis in patients with suspected GBM on MRI and for longitudinal monitoring of disease status. However, its utility for recurrence surveillance and for distinguishing pseudoprogression from true tumor progression after chemoradiotherapy requires prospective validation [25]. Moreover, CSF appears to provide a more consistent biofluid source for EV-miR-21 detection compared with serum, where results have shown greater variability across studies [25,56].

In serum EVs, levels of miR-21, miR-222, and miR-124-3p were significantly elevated in GBM patients compared with healthy controls, with individual AUCs of 0.84, 0.80, and 0.78, respectively [56]. Their combination improved diagnostic performance to an AUC of 0.87 [56]. The same panel also showed potential to discriminate high-grade glioma (HGG) from low-grade glioma (LGG) or normal controls [57]. These dynamic serum signatures may provide additional non-invasive information on tumor grade and disease dynamics.

A seven-miRNA signature (miR-182-5p, miR-328-3p, miR-339-5p, miR-340-5p, miR-485-3p, miR-486-5p, and miR-543) was reported in a pilot study to distinguish GBM from healthy controls with an accuracy of 91.7% [58].

Promising results warrant caution because of the limited number of patients, cohort heterogeneity, and variations in sample preparation and miRNA measurement methodologies. Intratumor heterogeneity and pre-analytical issues further confound EV-miRNA signatures. These limitations underscore the need for further methodological harmonization and independent validation, as discussed in Section 6. Similar EV-miRNA biomarker strategies are also being explored in NDDs, although those studies remain largely exploratory.

### 4.2. Prognostic and Monitoring Biomarkers

In prognostic studies, CSF exosomal miR-21 was markedly upregulated in glioma patients and correlated with tumor spinal/ventricular metastasis and recurrence, while tissue miR-21 levels also correlated with tumor grade [55]. Conversely, serum exosomal miR-21 levels did not differ significantly between patients and controls [55].

Serum exosomal miRNAs (miR-21, miR-222, and miR-124-3p) exhibited a considerable reduction post-surgery, followed by a rebound upon recurrence, supporting their potential utility for dynamic monitoring of treatment response and disease status [56,57]. In isocitrate dehydrogenase (IDH)-wildtype GBM, established molecular markers such as O^6^-methylguanine-DNA methyltransferase (MGMT) promoter methylation status are determined at diagnosis and do not provide dynamic longitudinal information on treatment response; dynamic serum EV-miRNA profiles may therefore offer complementary non-invasive monitoring over the disease course [15,56,57], although prospective validation in molecularly defined cohorts is required. In contrast to its reduced levels in tumor tissues, increased expression of serum exosomal miR-454-3p was significantly correlated with poor prognosis and markedly decreased after surgical resection [59]. 

In a small cohort, postoperative levels of CSF exosomal miR-1246 were associated with tumor recurrence [35]. Exosomal miR-151a transfer has been implicated in TMZ sensitivity in drug-resistant GBM, and CSF-derived exosomal miR-151a may reflect chemoresistant status and have predictive value for treatment response [49].

These findings suggest that circulating EV-miRNA profiles may improve patient stratification and may support longitudinal monitoring of tumor burden. However, current biomarker panels remain insufficiently validated and require further standardization before broader clinical application [78]. Selected diagnostic, prognostic, and monitoring EV-miRNA biomarkers discussed in this review are summarized in Table 2.

## 5. Therapeutic Opportunities of EV-miRNAs in GBM

### 5.1. Therapeutic Approaches

#### 5.1.1. Restoration of Tumor-Suppressor miRNA

Many tumor-suppressive miRNAs, including miR-124, are frequently downregulated in GBM and have been investigated as potential candidates for therapeutic restoration. As detailed in Section 3.2, MSC-derived exosomes carrying miR-124a achieved durable tumor suppression in a preclinical intracranial model, with 50% long-term survival and no residual tumor in survivors [41]. Separately, HEK293T-derived EVs loaded with miR-124 suppressed GBM cell proliferation and inhibited M2 microglial polarization in a 3D microfluidic model in vitro, as described in Section 3.2 [39]. Individual studies have also reported anti-GBM effects for EV-associated tumor-suppressive miRNAs such as miR-7, miR-145, miR-146b, miR-375, and miR-199a in preclinical models [82]. Collectively, the restoration of tumor-suppressive miRNAs has shown promising preclinical efficacy in GBM models.

#### 5.1.2. Inhibition of OncomiR

OncomiRs promote tumor cell progression and therapeutic resistance. EV-delivered miRNA sponges can inhibit oncomiRs [75]. Antisense oligonucleotides and genome editing offer alternative approaches [83].

EVs carrying an miR-21 sponge reduce cellular miR-21 levels and increase downstream tumor suppressor targets, such as PDCD4 (a translational repressor that inhibits cell proliferation and promotes apoptosis) and RECK (a membrane-anchored inhibitor of matrix metalloproteinases that suppresses tumor invasion and angiogenesis), in GBM cells, thereby suppressing proliferation and inducing apoptosis [75]. In a rat GBM model, exosomes packed with an miR-21 sponge inhibited tumor progression [75]. MSC-mediated delivery of anti-miR-9, with exosomes contributing to intercellular transfer, reversed the expression of multidrug transporters and sensitized GBM cells to TMZ [84]. Given GBM’s miRNA network complexity, multi-targeted miRNA sponges may be particularly relevant, as single-agent strategies have frequently shown limited efficacy [85,86]. Genome-editing tools, such as CRISPR/Cas9, offer sustained effects; however, translation is constrained by delivery and nonspecific uptake risks [87,88]. 

#### 5.1.3. Combination Therapeutic Approaches

Researchers have identified the combination of miRNA-based strategies, including EV-mediated delivery where applicable, with chemotherapy or immunotherapy as a promising approach for mitigating GBM resistance in preclinical models [89,90]. The co-delivery of miR-124 and PD-1 plasmids by umbilical cord mesenchymal stem cell (UMSC)-derived exosomes results in significant tumor inhibition and immune regulation in GBM models [91]. 

In a non-EV study, combinatorial delivery of the engineered miR-124/128/137 cluster with TMZ resulted in a 5-fold increase in survival in murine GBM models, through synchronized targeting of oncogenic chromatin repressors EZH2, BMI1, and LSD1, which drive therapy resistance and tumor recurrence post-chemoradiation [92]. This result provides a conceptual rationale for EV-based polycistronic miRNA delivery. Consistent with this concept, engineered exosomes co-delivering miR-124-2/135a-2/let-7i prolonged survival in orthotopic GBM models [93]. Taken together, combinatorial strategies may improve efficacy via multi-pathway interactions, addressing GBM heterogeneity and resistance, as elaborated in Section 6.

### 5.2. Engineering EVs for Enhanced Delivery

Engineering EVs for enhanced delivery is rapidly advancing and represents a promising approach to improve the therapeutic applications of miRNAs in GBM [94,95]. The engineered EV architectures described in this section should be understood as conceptual platforms validated in preclinical models, and their translation will require resolution of manufacturing, immunogenicity, and regulatory challenges outlined in Section 5.3. EVs have been modified to enhance cargo loading, target specificity, and BBB permeability [96,97]. 

#### 5.2.1. Cargo Loading Strategies

Endogenous loading strategies have been engineered to enable producer cells to package cargo during EV biogenesis while maintaining EV membrane integrity [61]. For example, the CD9–HuR fusion system enriched miR-155 in exosomes by fusing the exosomal membrane protein CD9 with the RNA-binding protein HuR, which was efficiently delivered to recipient cells and recognized endogenous targets both in vitro and in vivo [98].

Exogenous loading methods, such as electroporation, sonication, and freeze–thaw cycling, can load cargo into pre-isolated EVs and offer substantial flexibility; however, they are frequently associated with vesicle aggregation and variable loading efficiency [99].

Recent improvements in electroporation processes and polymer-assisted approaches have enhanced miRNA loading efficiency [94,99]. However, loading efficiencies vary, and standardized potency assays are required for reproducibility [99]. 

#### 5.2.2. Surface Engineering for Targeted Delivery

To achieve tumor-specific delivery, EV surfaces can be modified for tissue-specific targeting by genetically fusing targeting peptides to Lamp2b, CD63, or CD9 or by using approaches such as click chemistry (azide/dibenzocyclooctyne (DBCO) cycloaddition), liposome fusion, and nanobody or HaloTag display systems [94,100]. Ligands utilized for targeting include cRGD (an αvβ3 integrin-binding peptide that promotes tumor-targeted cellular uptake), aptamers (such as anti-EGFR), and antibodies (such as anti-PD-L1) [95,100]. 

#### 5.2.3. Improving BBB Penetration

Unmodified EVs can cross the BBB to a limited and context-dependent extent; however, adding BBB-targeting shuttle peptides can significantly boost their accumulation in the CNS [101]. The rabies virus glycoprotein (RVG) is widely used as a brain-targeting peptide and has been proposed to interact with nicotinic acetylcholine receptor-related pathways [102,103]. Angiopep-2 binds to low-density lipoprotein receptor-related protein 1 (LRP-1), which is overexpressed in BBB endothelial cells and glioma cells, supporting BBB and glioma targeting [104].

Cell-penetrating peptides (CPPs), including the trans-activator of transcription (TAT) peptide, facilitate cellular uptake and tissue penetration, thereby facilitating EV access to the brain parenchyma [105].

Dual-engineered sEVs functionalized with Angiopep-2 and TAT peptides have demonstrated improved glioma targeting and therapeutic efficacy over unmodified controls in preclinical models [106], providing a conceptual framework for targeted dual-peptide sEV-cargo delivery in glioma; the translational challenges of such platforms are discussed in Section 6.

### 5.3. Challenges in Preclinical and Translational Research

Several preclinical and translational challenges must be addressed before EV-miRNA-based therapies can become viable treatment options for GBM.

EV-miRNA stability varies with storage conditions. Temperature shifts, freeze–thaw cycles, and buffer choice affect cargo levels and EV structure, thereby lowering reproducibility in liquid biopsy studies [107,108].

Safety and immunogenicity are additional critical factors. Immune responses or adverse effects caused by delivery vehicles or modified EVs may reduce therapeutic efficacy. Modified or allogeneic EV-based systems may trigger the complement system or provoke cytokine release, while the mononuclear phagocyte system may swiftly eliminate circulating EVs [109].

The origin, administration method, and surface modifications of EVs affect their biodistribution [110], whereas regulatory evaluation remains challenging due to technical variations in the isolation, characterization, and quality monitoring of EV-miRNAs [64]. Good Manufacturing Practice (GMP)-compatible standardization for GBM-focused EV-miRNA therapeutics remains limited, and uncertainty regarding long-term safety in the CNS immune environment persists [64,109]. These manufacturing challenges have prompted interest in synthetic alternatives such as lipid nanoparticles and liposome-based carriers, which offer advantages in scalability and batch-to-batch consistency but lack the inherent biocompatibility and selective cargo properties of EVs; a detailed comparison falls outside the scope of this review.

## 6. Conclusions and Future Perspectives

This review summarizes the functions of EV-miRNAs in GBM, including their biogenesis, secretion, uptake mechanisms, and potential use as liquid biopsy biomarkers and therapeutic targets.

Notably, EV-miR-21 and EV-miR-124 are dysregulated in both GBM and selected NDD-related contexts, suggesting partially conserved mechanisms, such as microglial reprogramming and inflammation, which may inform shared diagnostic or therapeutic strategies across CNS disorders [20,111].

**Figure 1:**
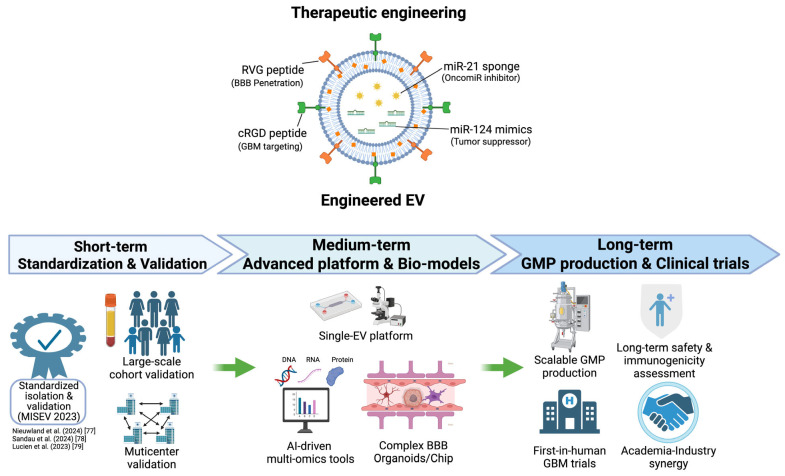
Conceptual overview of engineered EV strategies for miRNA-based therapy in GBM and the proposed multiphase roadmap for clinical translation [77,78,79]. Upper panel: This figure presents a conceptual schematic, not a validated therapeutic product. Engineered EVs surface-modified with RVG peptide for BBB penetration and cRGD peptide for GBM targeting co-deliver miR-124 mimics (tumor-suppressive miRNA) and miR-21 sponges (oncomiR inhibitor), thereby potentially promoting tumor suppression, TMZ sensitization, and favorable tumor immune microenvironment remodeling in preclinical models. Lower panel: Short-term priorities focus on MISEV2023 standardization and large-scale multi-center validation. Medium-term efforts include the development of single-EV analysis platforms, multi-omics computational tools, and GBM-relevant BBB models. Long-term goals encompass scalable GMP-compliant manufacturing, comprehensive safety and biodistribution assessments, advancing toward first-in-human GBM trials, and academia–industry–regulatory body collaboration. Abbreviations: BBB, blood–brain barrier; cRGD, cyclic Arg-Gly-Asp peptide; EV, extracellular vesicle; GBM, glioblastoma; GMP, Good Manufacturing Practice; MISEV, Minimal Information for Studies of Extracellular Vesicles; RVG, rabies virus glycoprotein.

EV-miRNAs in blood and CSF show diagnostic and longitudinal monitoring promise (Table 2) [23]. However, as noted in Section 4, most EV-miRNA biomarker studies remain in the preclinical or early clinical validation phase, with only a few signatures having entered small-scale prospective cohorts. These findings should be interpreted within the current WHO 2021 CNS tumor classification and in conjunction with established markers such as MGMT promoter methylation and IDH status [15,96]. On the therapeutic side, restoration of tumor-suppressor miRNAs (e.g., EV-carried miR-124) appears among the more translationally advanced preclinical strategies due to relatively simpler engineering and encouraging preclinical survival data. Inhibition of oncomiRs using miRNA sponges and combinatorial approaches with TMZ or immunotherapy may be viewed as intermediate-stage strategies, while highly engineered dual-targeted platforms remain at an early preclinical stage. To our knowledge, no registered EV-miRNA-based therapeutic trial for GBM has been identified to date. Figure 1 presents a conceptual schematic proposed here, rather than a single previously validated therapeutic product, combining RVG (for BBB-directed delivery) and cRGD (for GBM targeting) surface modifications with the co-delivery of a miR-124 mimic and miR-21 sponge.

Although most individual EVs carry sub-stoichiometric miRNA loads, collective vesicle uptake and deliberate cargo enrichment in engineered EVs may achieve functionally relevant concentrations in recipient cells [68,99]. Important translational considerations also remain, including limited endogenous miRNA loading, the need for single-vesicle-resolved characterization, and the continued limitations of classical “exosomal markers” (CD9, CD63, and CD81) in complex biofluids [64,68,99]. Challenges in clinical translation are manifold and include, but are not limited to, the scalability of GMP production, lack of formal regulatory frameworks, and absence of GBM-specific trials to date, as well as CNS-relevant barriers such as the unique immunological environment of the CNS [7,64]. The optimal route of administration (systemic vs. local) remains undefined [7].

Overcoming these hurdles will require concerted efforts from academia, industry, and regulatory bodies. Central to this effort are standardized EV isolation and characterization according to MISEV2023 guidelines; large multicenter validation studies with harmonized pre-analytical protocols; and the development of fit-for-purpose regulatory pathways for EV-miRNA therapeutics in neuro-oncology [64,112]. Key questions remain regarding the optimal biofluid for EV-miRNA profiling (blood vs. CSF), the reliability of patient-derived BBB models, and the mechanistic basis for paradoxical EV-miRNA expression patterns. Integrated single-cell transcriptomic and EV-miRNA profiling may help elucidate these discrepancies [7].

In terms of research priorities, the most immediate needs are rigorous methodological harmonization, prospective biomarker validation, and scalable GMP-compatible manufacturing pipelines. Advances in single-EV profiling and improved patient-derived BBB models are anticipated to follow as enabling technologies mature. Ultimately, comprehensive safety assessment and first-in-human trials of EV-miRNA combinations will be required to translate preclinical promise into clinical benefit.

Progress in this area could enhance the reproducibility and precision of neuro-oncology and extend precision applications beyond GBM to other CNS malignancies and neurodegenerative diseases.

## Figures and Tables

**Table 1:** Summary of key EV-miRNAs in GBM: oncogenic vs. tumor-suppressive roles, and cross-disease links with NDDs.

miRNA	Role in GBM	Expression in GBM	Key Mechanisms/Targets in GBM	Cross-Disease Links with NDDs (AD/PD/ALS)	References
miR-21	Oncogenic	↑	Promotes endothelial cell proliferation and VEGF/VEGFR2-driven angiogenesis; promotes MDSC-mediated immunosuppression via PTEN/PI3K/AKT pathway; exacerbates TMZ resistance via STAT3 activation in GAMs	Elevated in AD-related neuronal exosomes; propagates through neuron–microglia EV chain and induces pro-inflammatory microglial activation in vitro; MSC-derived exosomes carrying miR-21 reduce amyloid-β burden and neuroinflammation in AD mouse model; no robust EV-miR-21-specific data available in human AD. In PD, downregulated in serum EVs (correlative); upregulated in MPP^+^ dopaminergic neurons (non-EV, in vitro); EV-specific therapeutic evidence in PD remains lacking	[25,26,27,28,29,30,31,32]
miR-221	Oncogenic	↑	Targets DNM3; induces tumor progression and TMZ resistance in recipient cells	—	[33]
miR-1246	Oncogenic	↑	Targets TERF2IP; activates STAT3, suppresses NF-κB; promotes M2 macrophage polarization under hypoxia; drives MDSC differentiation and activation from CSF/plasma EVs; promotes cell migration and invasion via FRK targeting	—	[34,35,36]
miR-10b-5p	Oncogenic	↑	Abundant in hypoxic glioma-derived EVs; targets TFAP2A; promotes migration and invasion in recipient normoxic glioma cells	—	[36]
miR-124	Tumor-suppressive	↓	Suppresses STAT3 (M2→M1 microglia shift); reduces CDK6; silences FOXA2 (lipid metabolism in GSCs); enhances TMZ sensitivity; suppresses proliferation and migration in 3D microfluidic model; BM-MSC-derived exosomes overexpressing miR-124a achieved 50% long-term survival in intracranial xenograft model	Reduced in AD-related serum/CSF EVs; implicated in APP/BACE1 axis and tau phosphorylation; engineered EV-miR-124-3p shows neuro- and immunoprotective effects in AD models. In PD, total circulating miR-124 reduced (non-EV background); EV-miR-124-3p protects dopaminergic neurons in 6-OHDA model. In ALS, reduced in SOD1G93A spinal cord; motor neuron-derived EV cargo potentially including miR-124 associated with microglial NF-κB activation and impaired phagocytosis; therapeutic EV-miR-124 in ALS remains lacking	[29,37,38,39,40,41,42,43,44,45,46,47,48,49,50,51,52]
miR-1	Tumor-suppressive	↓	Targets ANXA2; loading into GBM-derived EVs reduces invasion, neurosphere formation, and endothelial tube formation	—	[53]
miR-199a	Tumor-suppressive	↓	Downregulates AGAP2; MSC-derived exosomal delivery suppresses glioma cell proliferation, migration, and invasion	—	[54]

Abbreviations: AD, Alzheimer’s disease; AGAP2, Arf GTPase-activating protein-2; AKT, protein kinase B; ALS, amyotrophic lateral sclerosis; ANXA2, Annexin A2; APP, amyloid precursor protein; BACE1, beta-site APP cleaving enzyme 1; BM-MSC, Bone Marrow-derived Mesenchymal Stem Cells; CDK6, cyclin-dependent kinase 6; CSF, cerebrospinal fluid; DNM3, dynamin 3; EV, extracellular vesicle; FOXA2, forkhead box protein A2; FRK, Fyn-related kinase; GAM, glioma-associated microglia/macrophage; GBM, glioblastoma; GSC, glioma stem cell; 6-OHDA, 6-hydroxydopamine; MDSC, myeloid-derived suppressor cell; MSC, mesenchymal stem cell; NDD, neurodegenerative disease; NF-κB, nuclear factor kappa B; PD, Parkinson’s disease; PI3K, phosphoinositide 3-kinase; PTEN, phosphatase and tensin homolog; SOD1G93A, superoxide dismutase 1 glycine-93-alanine mutant; STAT3, signal transducer and activator of transcription 3; 3D, three-dimensional; TERF2IP, telomeric repeat-binding factor 2-interacting protein; TFAP2A, transcription factor AP-2 alpha; TMZ, temozolomide; VEGF, vascular endothelial growth factor; VEGFR2, vascular endothelial growth factor receptor 2. ↑, upregulated; ↓, downregulated; —, not reported in this review.

**Table 2:** Selected EV-miRNA diagnostic, prognostic, and monitoring biomarkers in GBM discussed in this review.

miRNA/Signature	Biofluid	Study Type	Diagnostic Value	Prognostic/Monitoring Value	References
miR-21	CSF	Clinical	AUC 0.91, sensitivity 87%, specificity 93%; ~10-fold higher in GBM vs. non-tumor controls	Correlates with tumor spinal/ventricular metastasis and recurrence	[25,55]
miR-21, miR-222, miR-124-3p (combination)	Serum	Clinical prospective	Individual AUCs: miR-21 0.84, miR-222 0.80, miR-124-3p 0.78; combined AUC 0.87; discriminates HGG from LGG or normal controls	Dynamic monitoring of treatment response and disease status	[56,57]
7-miRNA signature (miR-182-5p, miR-328-3p, miR-339-5p, miR-340-5p, miR-485-3p, miR-486-5p, miR-543)	Serum	Clinical pilot	Accuracy 91.7% distinguishing GBM from healthy controls	—	[58]
miR-454-3p	Serum	Clinical	—	Elevated levels correlate with poor prognosis; markedly decreased after surgical resection	[59]
miR-1246	CSF	Clinical (small cohort)	—	Postoperative levels associated with tumor recurrence	[35]
miR-151a	CSF	Clinical	—	Exosomal miR-151a implicated in TMZ sensitivity; CSF-derived levels may reflect chemoresistant status and predict treatment response	[60]

Abbreviations: AUC, area under the curve; CSF, cerebrospinal fluid; GBM, glioblastoma; HGG, high-grade glioma; LGG, low-grade glioma; TMZ, temozolomide. —, not reported in this review.

## Data Availability

No new data were created or analyzed in this study. Data sharing is not applicable to this article.
